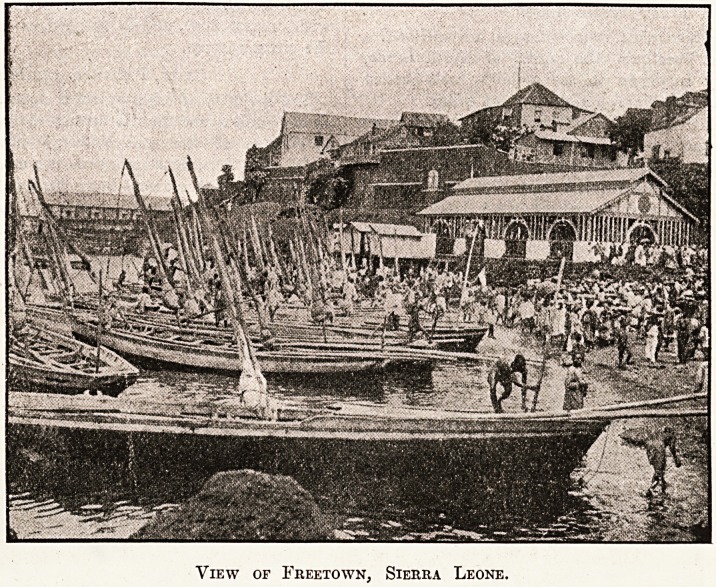# The Military Hospital, Sierra Leone

**Published:** 1915-05-29

**Authors:** 


### THE MILITARY HOSPITAL, SIERRA LEONE.

BY ONE RECENTLY ON THE STAFF.

WHEN I volunteered for foreign service and was given a post at the Military Hospital at Sierra Leone I began, of course, to try to find out what sort of place it was. I imagined it to be a hot, marshy jungle, all swamps and mangroves and alligators.

### FIRST IMPRESSIONS.

When, after a somewhat rough passage, we anchored a mile off shore at Freetown, the capital of Sierra Leone, I was agreeably surprised to see a hilly, well-wooded country, with apparently not a swamp for miles.

We disembarked by means of small boats, and were landed on the quay, and after the usual excitement of collecting luggage, etc., we started for the hospital, which is situated half a mile from the quay and half-way up Tower Hill, about 200 feet above sea-level. After a warm walk through strange and interesting scenes we reached our destination, and were warmly welcomed by the staff, whom we had come to relieve. We were not cheered by their appearance; they were yellow and emaciated, having been especially unfortunate with regard to malaria. Still, there we were for twelve months, willy nilly.

THE MILITARY HOSPITAL, SIERRA LEONE.

### THE INSTITUTION.

The hospital, which serves the greater part of the West Coast of Africa, is a large oblong brick and plaster building, with walls about three feet thick, numerous windows and doors, and a verandah round the two upper floors. It stands in its own compound, which is surrounded by a fence of iron spikes, and is planted with many kinds of flowering evergreens, etc., which, with mango and other trees, make the place look very attractive.

The ground floor of the hospital is used for medical, linen, and steward's stores. The first floor is one big ward of twenty-five beds for general cases. The second floor is divided into two wards, one for malaria and one for special cases, and is usually the coolest spot in the hospital. In addition, there is a block containing about twenty beds built especially for natives and West Indians requiring operations. The operating theatre, which is situated a little apart from the main building, is of brick with cement floor, and is as good as can be achieved in the circumstances.

### CASES, PATIENTS, AND STAFF.

The majority of cases operated upon are climatic bubos among whites and hernia among the natives. A large percentage of the native troops have hernia, chiefly umbilical. All important operations among whites (except emergency ones) are sent home for treatment. The black troops nursed are the West African Regiment, recruited on the spot, and the West Indian Regiment, brought from Jamaica and relieved every three years. They recover very quickly, and are sometimes found on the verandah the day after operation.

Diet consists of bread, milk, and rice. Pulmonary disease is unknown. Malaria is rife, sometimes a percentage of $2\frac{1}{2}$ being counted among the troops. There are also many cases of black-water fever, some malarial coma, insanity caused by sunstroke, and occasional snake and scorpion bites. The staff includes a medical officer, dispenser, and skiagraphist, and two or three orderlies. No females are allowed on account of the climate. "Black boys" do most of the rougher work, under the direction of the staff, and are fairly intelligent.

Opposite the hospital are the headquarters offices, set in a piece of waste ground, which during the rainy season is covered with tall coarse grass. Three or four miles to the west the Wilberforce Ridge rises. This well-wooded ridge is about four miles in length, and commences at Signal Hill on the bank of the Rochelle River and ends at Hill Station. Between the Ridge and the hospital is crowded a part of Freetown, consisting mainly of wooden houses and grass huts.

Climate.

The climatic conditions are fixed; the hot season, from November to February, is entirely rainless. Temperature from 90° to 100° in the shade and 130° to 140° in the sun. The rainy season lasts the rest of the year. This starts with brief storms, then there is continual rain for two or three months, then storms again. As is usual in tropical countries, there is no twilight. Black, velvety darkness settles down about 6 p.m., leaving a long evening to be worried through, broken only by the buzz of the mosquitoes and the everlasting drone of tom-toms from the native huts.

During the rainy season vegetation flourishes in an astonishing degree. The grass grows breast-high in about six weeks. Malaria also flourishes, and to prevent it a most careful look-out is kept for broken bottles, tins, etc., which allow a breeding-place for the larvæ of mosquitoes. Even holes in trees and rocks are cemented up, and pools of stagnant water are disinfected or covered with a layer of oil whenever possible.

During fine weather, if one can stand the heat, some beautiful walks can be taken. Gorgeous coloured flowers and birds and butterflies abound, and the scenery about Sugar Loaf Mountain, for instance, which is four or five miles from Freetown and rises to about 2,000 feet, is very impressive.

View of Freetown, Sierra Leone.

## Figures and Tables

**Figure f1:**
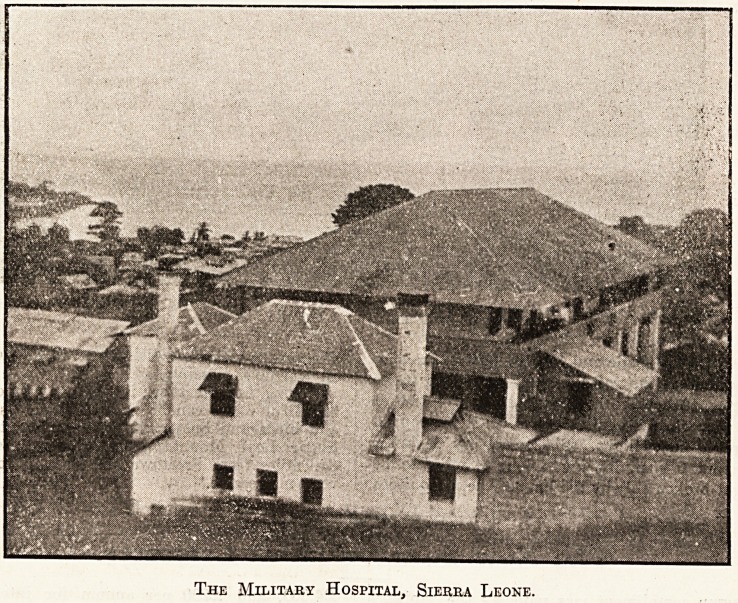


**Figure f2:**